# Acceleration of Age-Associated Methylation Patterns in HIV-1-Infected Adults

**DOI:** 10.1371/journal.pone.0119201

**Published:** 2015-03-25

**Authors:** Tammy M. Rickabaugh, Ruth M. Baxter, Mary Sehl, Janet S. Sinsheimer, Patricia M. Hultin, Lance E. Hultin, Austin Quach, Otoniel Martínez-Maza, Steve Horvath, Eric Vilain, Beth D. Jamieson

**Affiliations:** 1 Department of Medicine, Division of Hematology/Oncology, AIDS Institute, University of California Los Angeles, Los Angeles, California, United States of America; 2 Department of Human Genetics, University of California Los Angeles, Los Angeles, California, United States of America; 3 Biomathematics, David Geffen School of Medicine, University of California Los Angeles, Los Angeles, California, United States of America; 4 Department of Biostatistics, School of Public Health, University of California Los Angeles, Los Angeles, California, United States of America; 5 Department of Epidemiology, School of Public Health, University of California Los Angeles, Los Angeles, California, United States of America; 6 Departments of Obstetrics and Gynecology, and Microbiology, Immunology and Molecular Genetics, University of California Los Angeles, Los Angeles, California, United States of America; Emory University, UNITED STATES

## Abstract

Patients with treated HIV-1-infection experience earlier occurrence of aging-associated diseases, raising speculation that HIV-1-infection, or antiretroviral treatment, may accelerate aging. We recently described an age-related co-methylation module comprised of hundreds of CpGs; however, it is unknown whether aging and HIV-1-infection exert negative health effects through similar, or disparate, mechanisms. We investigated whether HIV-1-infection would induce age-associated methylation changes. We evaluated DNA methylation levels at >450,000 CpG sites in peripheral blood mononuclear cells (PBMC) of young (20-35) and older (36-56) adults in two separate groups of participants. Each age group for each data set consisted of 12 HIV-1-infected and 12 age-matched HIV-1-uninfected samples for a total of 96 samples. The effects of age and HIV-1 infection on methylation at each CpG revealed a strong correlation of 0.49, p<1 x10^-200^ and 0.47, p<1x10^-200^. Weighted gene correlation network analysis (WGCNA) identified 17 co-methylation modules; module 3 (ME3) was significantly correlated with age (cor=0.70) and HIV-1 status (cor=0.31). Older HIV-1^+^ individuals had a greater number of hypermethylated CpGs across ME3 (p=0.015). In a multivariate model, ME3 was significantly associated with age and HIV status (Data set 1: β_age_= 0.007088, p=2.08 x 10^-9^; β_HIV_= 0.099574, p=0.0011; Data set 2: β_age_= 0.008762, p=1.27x 10^-5^; β_HIV_= 0.128649, p= 0.0001). Using this model, we estimate that HIV-1 infection accelerates age-related methylation by approximately 13.7 years in data set 1 and 14.7 years in data set 2. The genes related to CpGs in ME3 are enriched for polycomb group target genes known to be involved in cell renewal and aging. The overlap between ME3 and an aging methylation module found in solid tissues is also highly significant (Fisher-exact p=5.6 x 10^-6^, odds ratio=1.91). These data demonstrate that HIV-1 infection is associated with methylation patterns that are similar to age-associated patterns and suggest that general aging and HIV-1 related aging work through some common cellular and molecular mechanisms. These results are an important first step for finding potential therapeutic targets and novel clinical approaches to mitigate the detrimental effects of both HIV-1-infection and aging.

## Introduction

Aging is associated with an increasing incidence of chronic, debilitating, diseases. While cardiovascular, skeletal, and neurodegenerative diseases are widely known and discussed in the general population, there is virtually no organ or tissue system that is not at risk. The mechanisms underlying aging and its deleterious effects are poorly understood, but thought to be multifactorial and to involve epigenetic changes [1]. Epigenetics is the alteration of DNA through modifications that do not change the underlying nucleotide sequence [2], yet are important in controlling gene expression. There are many types of epigenetic regulation, including small RNAs, acetylation of histones and the methylation of cytosine residues [3]. Currently available array and sequencing technology has allowed genome-wide examination of methylation levels, and there now exists a body of literature showing both global and site-specific changes in methylation patterns in relation to age [4–14].

There are several lines of evidence to suggest that HIV-1-infection accelerates at least some aspects of the aging process [15–16]. Perhaps the most dramatic piece of evidence is that successfully treated HIV-1-infection is associated with a greater susceptibility to morbidities more commonly observed in older, uninfected, individuals such as frailty [17], non-Hodgkin's lymphoma [18], anal and cervical carcinomas [19–20], osteoporosis [21–22], liver [23–25] and renal impairment [26], cardiovascular disease [27–28], diabetes [29] and hypertension [29–30]. There is also evidence of faster disease progression in older HIV-1-infected adults [31].

As we have documented, HIV-1-infection is also associated with aging-related changes in the CD4^+^ T-cell compartment [32]. There is a significant decrease in the number of CD31^+^ naïve CD4^+^ T-cells and shortening of telomeres in the overall naïve CD4^+^ T-cell subpopulation, rendering this cellular compartment more phenotypically similar to that of an uninfected adult 20–30 years older [32]. There is also evidence of telomere shortening within CD8^+^ T-cells and a significant increase in senescent CD8^+^ T-cells in HIV-1-infected individuals, similar to that observed in older seronegative individuals [33–34]. Together, these findings have led to the suggestion that HIV-1-infection and aging may interact in a mechanistic manner.

To date, the vast majority of data examining the interrelationship between HIV-1-infection and aging have been obtained at the cellular and organismal level. Much of these data measure outcomes and are unable to address mechanisms or pathways. For example, the finding that HIV-1-infection renders individuals more likely to develop frailty 10 years earlier than their uninfected peers [17] is an outcome. What is missing are data regarding the mechanism(s) by which HIV-1-infection contributes to the earlier manifestation of age-inappropriate clinical outcomes and how those mechanisms overlap with, or are disparate from, the mechanisms responsible for aging-associated comorbidities in the absence of HIV-1-infection.

Using a systems biologic analysis approach we recently revealed a robustly defined age-related co-methylation module that is present in multiple human tissues, including saliva [35], blood, and brain [12]. These studies demonstrate that blood is a promising surrogate for other tissues when studying the effects of age on DNA methylation profiles [12,14] and that the aging module could be an important biomarker for detecting accelerated aging effects.

We used two unique data sets to carry out a weighted correlation network analysis (WGCNA) of DNA methylation data and identified an age related co-methylation module, or aging module, that contains CpG sites that are hypermethylated with age. This is the first study that shows that an age-related co-module, a new biomarker of aging, detects accelerated aging epigenetic effects due to HIV infection. We also show that our systems biologic analysis based on WGCNA leads to more pronounced biological insights than a standard differential methylation analysis that only considers marginal relationships between CpG sites and HIV infection. Comparison of this module to our previously found aging module [12] revealed that it can also be found in other solid tissues, notably human brain tissue, and may thus also measure organismal aging effects. These unique tools may aid in the elucidation of novel therapeutic targets for aging-related clinical diagnoses in HIV-infected and uninfected individuals.

## Methods

### Ethics Statement

This study was approved by the University of California, Los Angeles Medical Institutional Review Board and each participant was provided written, informed consent per the approved protocol-IRB# 10–001677.

### Participants

We selected participants from the Multicenter AIDS Cohort Study (MACS), a study of the natural and treated history of HIV-1 infection in men who have sex with men [36]. There were two groups of participants in data set one: 24 of the samples were from individuals 20–24 years of age and 24 were from individuals 48–56 years of age. In each group, 12 of the samples were from HIV-1 seropositive (SP) men and 12 were from HIV-1 seronegative (SN) men. There were two groups of participants in data set two: 24 of the samples were from individuals 27–35 years of age and 24 were from individuals 36–56 years of age. In each group, 12 of the samples were from HIV-1 SP men and 12 were from HIV-1 SN men. Selection criteria included the following characteristics: age, visit date, anti-retroviral treatment (ART) naïve (self-reported during the semi-annual MACS study visits), history of smoking, and ethnicity. Each HIV-1 seropositive sample was individually matched to a seronegative control using the selection criteria.

We examined BMI data on our participants and we do not find any significant difference in BMI for our HIV-1 seronegative and HIV-infected participants. The seronegative participants had an average BMI of 23.9 and a median BMI of 23.8. The seropositive participants had an average BMI of 22.4 and a median BMI of 22.2. We also examined chronic co-infection with Hepatitis B and C, two common viruses that are known to cause chronic infection. In our first group of samples, two of the HIV-1-infected participants had chronic Hepatitis C and two of the HIV-1-infected participants had chronic Hepatitis B infection (none of the seronegatives in either group had a chronic infection).

### DNA Isolation

Human peripheral blood mononuclear cell (PBMC) samples were isolated from fresh blood samples and either stained for flow cytometry analysis or used for genomic DNA isolation. DNA was isolated from 1x10^6^ PBMC using Qiagen DNeasy blood and tissue mini spin columns. Quality of DNA samples was assessed using nanodrop measurements and accurate DNA concentrations were measured using a Qubit assay kit (Life Technology).

### Flow Cytometry

Cryopreserved PBMC obtained from the Multi-Center AIDS Cohort Study (MACS) repository were thawed and assayed for viability using trypan blue. The mean viability of the samples was 88%. Samples were stained for 30 minutes at 4°C with the following antibody combinations of fluorescently conjugated monoclonal antibodies using the manufacturers recommended amounts for 1 million cells: tube 1) CD57 FITC (clone HNK-1), CD28 phycoerythrin (PE, L293), CD3 peridinin chlorophyll protein (PerCP,SK7), CD45RA phycoerythrin cyanine dye Cy7 tandem (PE-Cy7, L48), CCR7 Alexa Fluor 647 (AF647, 150503), CD8 allophycocyanin H7- tandem (APC-H7, SK1) and CD4 horizon V450 (V450, RPA-T4), tube 2) HLA-DR FITC (L243), CD38 PE (HB7), CD3 PercP, CD45RO PE-Cy7 (UCHL-1), CD95-APC(DXZ), CD8 APC-H7, and CD4 V450), tube 3) CD38 FITC (HB7), IgD PE (1A6–2), CD3 PerCP, CD10 PE-Cy7 (HI10a), CD27 APC (eBioscience, clone 0323, San Diego, CA), CD19 APC-H7 (SJ25C1) and CD20 V450 (L27). Antibodies were purchased from BD Biosciences, San Jose, CA (BD) except as noted. Stained samples were washed twice with staining buffer and run immediately on an LSR2 cytometer equipped with a UV laser (BD, San Jose, CA) for the detection of 4’,6-diamidino-2-phenylindole dihydrochloride (DAPI) which was used as a viability markers at a final concentration 0.1 ug/ml. Lineage gated isotype controls to measure non-specific binding were run and used CD3, CD4 and CD8 for T-cells or CD19 for B-cells. Fluorescence minus one controls (FMO) were also utilized to assist gating and cursor setting. 20,000 to 100,000 lymphocytes were acquired and analyzed per sample using the FACSDiva software package (BD, San Jose).

### Methylation Arrays

Methylation status, at over 450,000 CpG sites, was measured to single-base resolution using Infinium methylation 450 arrays. These arrays interrogate methylation sites covering 99% of RefSeq genes with an average of 17 CpG sites throughout the promoter 5’ and 3’ UTRs and coding regions of each gene. In addition the arrays cover CpG islands, island shores and other sites distributed throughout the genome. Genomic DNA was prepared as described above. For both data sets, each SP sample had a matched SN control that was placed on the same chip. Samples were divided so that each chip contained 3 paired samples from the younger age group and 3 paired samples from the older age group. Within each chip the samples were arranged so that SP samples were not placed in adjacent spots with their matched SN controls, the older and younger samples were alternated, and one of each type of sample on the chip occupied each corner.

Microarray hybridization was performed by the Southern California Genotyping Consortium at UCLA. 500 ng of genomic DNA was bisulfate converted using the EZ-methylation kit (Zymo Research). The chips were processed using the Illumina Infinium whole genome genotyping protocol. Labeled samples were hybridized to the Illumina Human-Methylation450 arrays, scanned (iScan reader, Illumina), and β (methylation) values were obtained using GenomeStudio software. We followed the standard protocol of Illumina methylation assays, which quantifies methylation levels by the β value using the ratio of intensities between methylated (signal B) and unmethylated (signal A) alleles. Specifically, the β value was calculated from the intensity of the methylated (M corresponding to signal A) and unmethylated (U corresponding to signal B) alleles, as the ratio of fluorescent signals β = Max(M,0)/[Max(M,0) +Max(U,0) + 100]. Thus, β values range from 0 (completely unmethylated) to 1 (completely methylated).

The Illumina 450K platform uses two different chemical assays. The Infinium I and Infinium II assays for the assessment of the DNA methylation status of more than 480,000 cytosines distributed over the whole genome. Teschendorff et. al developed a model-based intra-array normalization strategy for the 450K platform, called BMIQ (Beta Mixture Quantile dilation), which adjusts beta-values of type II probes into a statistical distribution characteristic of type I probes [37]. We used the background corrected beta value values and subsequently the BMIQ normalization procedure. We did not filter out any CpGs since our module based analysis is unaffected by possibly misannotated CpG sites.

### Weighted correlation network analysis for finding co-methylation modules

Correlation network methodology has been widely used for studying relationships between gene transcripts. Recently, we and others have shown that these techniques are also useful for studying relationships between the DNA methylation levels of CpGs [12,35]. To describe the relationships among methylation profiles, we used a widely used approach: weighted correlation network analysis (WGCNA) [38]. Since prior work had shown that CpGs with a positive age relationships have a different biological interpretation than negatively correlated CpGs [12], we used signed weighted correlation network analysis that leads to co-methylation modules comprised of positively related CpGs. The goal of our WGCNA analysis was i) to identify modules, and ii) to calculate a representative of each module (module eigenvectors), iii) to correlate module eigenvectors with age and HIV1 infection status, and iv) to define a continuous measure of module membership in the consensus module (referred to as kME).

To define modules (clusters) we used average linkage hierarchical clustering with the topological overlap based dissimilarity measure because it is a highly robust measure that compares favorably with other distance measures in the case of genomic data [39–40]. Modules, branches of the resulting clustering tree, were subsequently identified using the dynamic hybrid branch cutting approach implemented in the R package dynamicTreeCut [41]. Because each module groups together highly correlated methylation profiles, it is useful to summarize the profiles in each module using a single representative profile. Here we use the module eigenvector [42], defined as the first principal component of the module methylation matrix. For each module, its module eigenvector can be used to define a measure of module membership, denoted kME, which quantifies how close a methylation profile is to the module. Specifically, for each methylation profile and each module, kME is defined as the correlation of the methylation profile with the module eigenvector. Defining module membership as correlation allows one to easily calculate the statistical significance (P-value) of each module membership. Module membership measures allow one to efficiently annotate all 480k CpGs on the Illumina Infinium 450K array with respect to module membership [43]. CpGs with high kME values with respect to a particular module are informally referred to as intramodular hub CpGs.

### Module preservation statistics

An advantage of WGCNA is that it provides powerful module preservation statistics that assess whether the density (how tight interconnections among genes in a module are) and connectivity patterns of individual modules (for example, intramodular hub gene status) are preserved between two data sets [44]. To assess the preservation our modules from the first data set (reference network) in the test network (second HIV data sets), we used the R function ‘modulePreservation’ in the WGCNA R package, as this quantitative measure of module preservation enables rigorous argument that a module is not preserved [44]. By averaging the several preservation statistics generated through many permutations of the original data, a *Z*_summary_ value is calculated, which summarizes the evidence that a module is preserved and indicative of module robustness and reproducibility. In general, modules with *Z*_summary_ scores >10 are interpreted as strongly preserved (that is, densely connected, distinct, and reproducible modules), *Z*_summary_ scores between 2 and 10 are weak to moderately preserved, and *Z*_summary_ scores <2 are not preserved.

### Determination of Reproducibility of the Data Sets

Standard screening for HIV-CpG associations was conducted using unpaired two-tailed t-tests and q-value adjustment with the significance threshold of q < 0.05. The agreement of these associations was assessed between datasets using Pearson’s correlation.

### Evaluation using Reference Free EWAS

The Reference Free EWAS package developed by Houseman *et al*. was applied to our PBMC datasets to assess the influence of cell subset proportions on the association of HIV-CpG methylation associations [45]. Age and HIV status were used as covariates in generating the refFreeEWAS model, and the number of latent variables estimated by random matrix theory was 5 and 13 for data sets 1 and 2, respectively. Pearson’s correlation was used to determine the concordance between datasets before and after RefFreeEWAS adjustment.

## Results

### The effects of HIV-1 infection on changes in methylation are additive with the effects of aging

To investigate the effects of HIV-1 infection on methylation patterns and distribution of cell types in peripheral blood we obtained cryopreserved peripheral blood mononuclear cells (PBMC) from the Multicenter AIDS Cohort Study (MACS) [36]. For data set one, samples obtained were from younger (20–24 years) and older adults (48–56 years). Each group consisted of 12 HIV-1-infected individuals and 12 age-matched HIV-1 uninfected controls. For data set two, samples were obtained from a different group of younger (27–35 years) and older adults (36–56 years), with each group containing the same number of HIV-1-infected and uninfected samples as data set one. To avoid the confounding effects of anti-retroviral therapy (ART), all of the HIV-1-infected participants were ART naïve.

**Fig 1:**
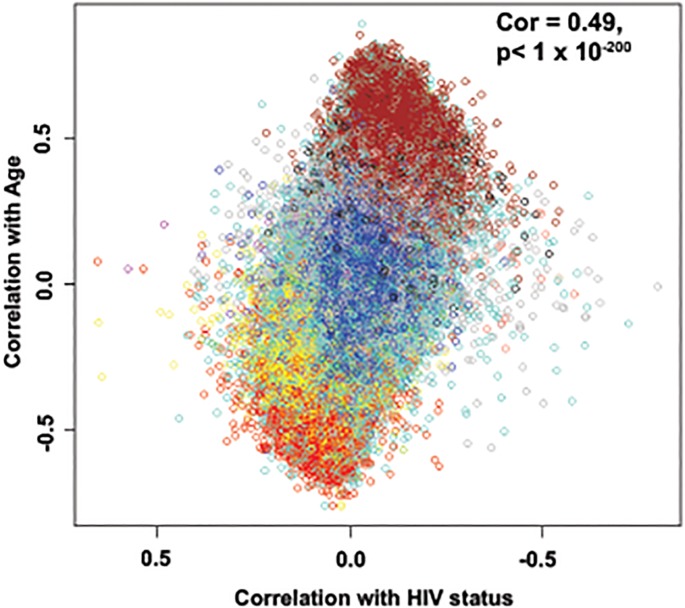
Age effects versus HIV-1 effects on methylation status. Methylation differences for each of the 24 pairs of samples were calculated and a paired t-test was performed for each of the CpG sites on the 450K array. The HIV-1 effect (X-axis) was measured as the signed logarithm of the Student t-test p-value. Age effects (Y-axis) were measured by the Pearson correlation coefficient with age. Each dot is colored according to its module membership (See Fig. 2). This is a representative figure for both data sets.

Methylation levels were measured at over 450,000 individual sites using Infinium methylation 450K arrays. The relationship between the effects of age and HIV-1 infection on changes in the degree of methylation at each site was assessed and revealed a strong correlation of 0.49, p<1 x10^-200^ for data set one (Fig. 1) and 0.47, p<1x10^-200^ for data set two (data not shown). Also, the reproducibility of the association of HIV status and methylation correlated significantly between both data sets (S1 Fig.). Interestingly, a subset of sites that are hypermethylated with age show a further increase in methylation in individuals infected with HIV-1, while a subset of sites hypomethylated with age was associated with earlier hypomethylation in HIV-1-infected individuals in both data sets.

**Fig 2:**
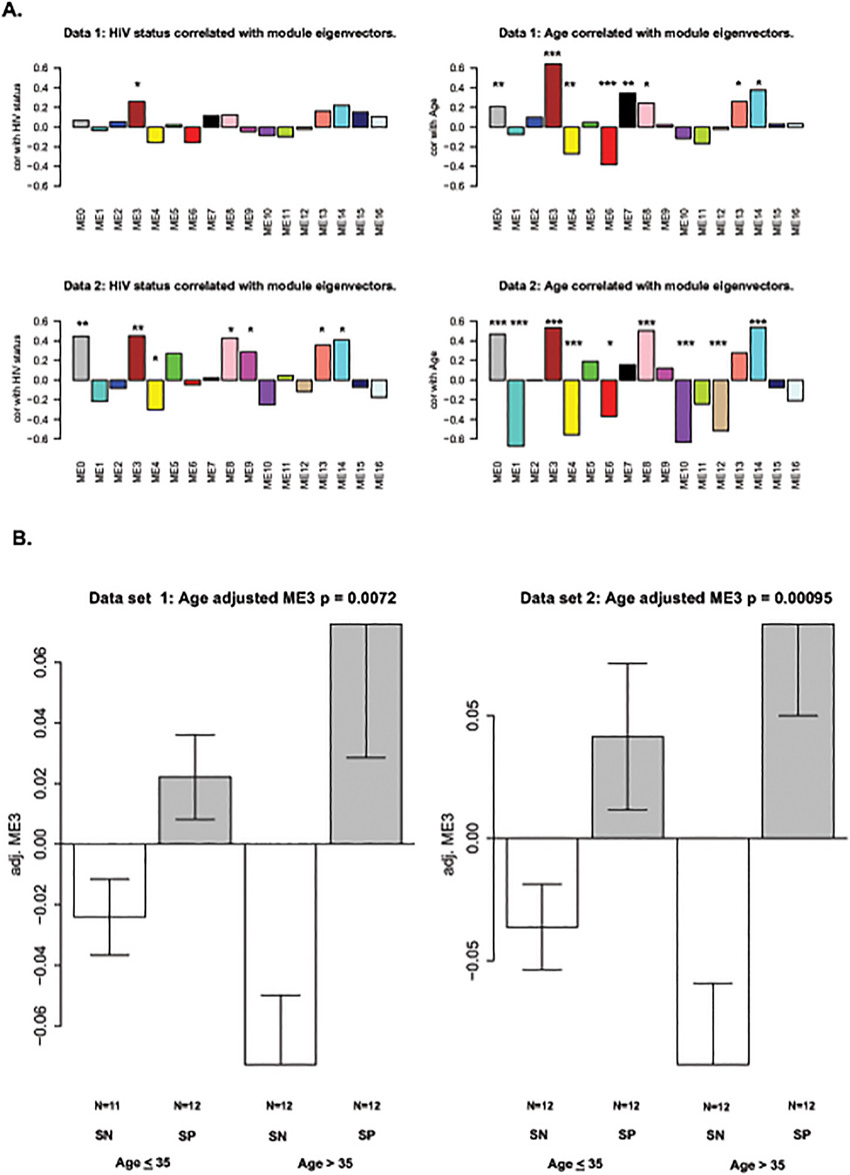
Relating modules to HIV-status and age. Co-methylation modules for HIV-1 status and aging (A) were identified using the blockwise modules function in WCGNA R package. The significant p values for the modules are indicated as follows: * = p≤0.05, ** = p≤0.01, *** = p≤0.001 (B) A box plot depicting module 3 versus age and HIV status.

### HIV-1 infection accelerates age related changes in methylation

We used the blockwise modules function in the WCGNA R package [38] to identify co-methylation modules in the methylation dataset. A co-methylation module is a cluster of CpGs that are correlated with each other across the samples analyzed. To determine the module preservation between the data sets, a Z_summary_ score was also obtained using the R function ‘modulePreservation’ in the WGCNA R package. This quantitative measure of module preservation has been shown to be a highly effective way to determine if a module is, or is not, preserved between data sets (Table 1) [44].

**Table 1:** Module preservation between data sets.

Module Number	Module Size (number of CpGs)	Z_summary_ *
**1**	**317,843**	**14.8**
**2**	**75,228**	**12.3**
**3**	**18,952**	**67.8**
**4**	**11,453**	**73.6**
**5**	**11,357**	**2.7**
**6**	**7,135**	**71.0**
**7**	**2,381**	**59.3**
**8**	**2,148**	**9.3**
**9**	**2,016**	**12.3**
**10**	**1,416**	**24.0**
**11**	**1,148**	**50.7**
**12**	**820**	**13.2**
**13**	**604**	**74.9**
**14**	**538**	**11.0**
**15**	**362**	**8.3**
**16**	**189**	**1.1**

*Z_summary_ > 10: module highly preserved

Z_summary_ > 5: module moderately preserved

Z_summary_ < 2: module not preserved

This analysis showed that the effects of HIV-1-infection and age (Fig. 2A) result in similar clustering patterns in both data sets, suggesting that similar methylation sites are affected by both age and HIV-1-infection. Module 3, ME3, showed the strongest positive correlation for both age and HIV-1 status. Module 3 is also highly preserved between both data sets with Z_summary_ scores of 67.8 (Table 1) [44]. Overall, the expression pattern of the module 3 eigengene across participant groups shows that CpGs of the module tend to be significantly hypermethylated in HIV+ subjects compared to HIV- subjects. The results are more pronounced for older subjects (Fig. 2B).

In a multivariate model examining associations between methylation, age and HIV status, module 3 was significantly associated with both age (data set one: p = 2.08 x 10^-9^; data set two: p = 1.27 x 10^-5^) and HIV-1 status (data set one: p = 0.0011; data set two: p = 0.0001) (Table 2). Using this model, we estimate that HIV-1 infection accelerates age-related methylation changes in peripheral blood mononuclear cells by an average of 13.7 years in data set one and 14.7 years in data set 2 (Table 2).

**Table 2:** Estimating accelerated aging due to HIV-1 infection using a multivariate model.

	Data set 1 Coefficients (SD)	Data set 1 Pr (>I t I)	Data set 2 Coefficients (SD)	Data set 2 Pr(>I t I)
**Intercept**	-0.3158663 (0.041)	1.15 x 10^-09^	-0.387857 (0.069)	1.24 x 10^-6^
**Age**	0.0070888 (0.009)	2.08 x 10^-09^	0.008762 (0.002)	1.27 x 10^-5^
**HIV Seropositive**	0.0969574 (0.028)	0.0011	0.128649 (0.031)	0.00014
Estimate of accelerated aging*	13.7 years		14.7 years	

*Using the output above, it is estimated that HIV status accelerates age by 13.7 and 14.7 years (defined by HIV coefficient/Age coefficient)

### The numbers of activated, memory, and senescent T-cells positively correlate with Module 3

Both aging and HIV-1-infection have been associated with similar changes within the lymphocyte compartment [46–47]. To assess whether these cellular changes are also associated with age or HIV-1-specific methylation patterns, the absolute numbers of various T and B cell subsets in the periphery were assessed using flow cytometry on an aliquot of cells from the same vial that yielded cells for methylation analysis. Cell surface markers known to differentiate naïve, memory, terminally differentiated, and activated T-cells, as well as immature/mature B-cells, were used.

**Fig 3:**
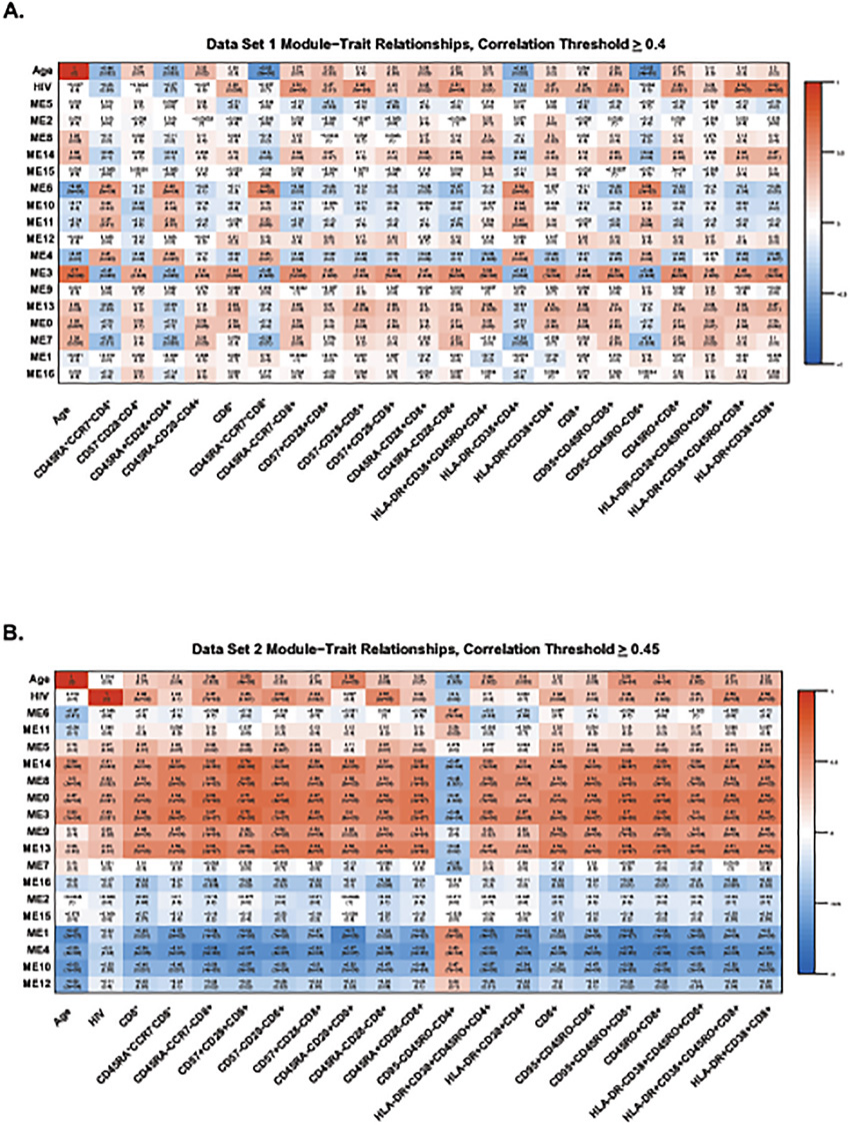
Heat map of module-trait relationships. This heat map shows correlations between HIV status, chronological age, and the co-methylation module (represented by their eigenvectors) for data set one (A) and data set two (B). Included are cell subsets whose absolute numbers have an absolute correlation with module 3 that was ≥0.4. Red depicts a positive correlation, blue depicts a negative correlation, as indicated by the color band on the right.

Using this flow cytometry data, we found that methylation status does correlate highly with absolute numbers of several T-cell subsets in both data sets. Fig. 3 shows the module-trait relationships between different T-cell subsets identified by flow cytometry and each module with a correlation value of ≥0.4. In addition to correlating with HIV-1 status and age, many T-cell subsets are also strongly positively correlated (correlation coefficient ≥ 0.34) with module 3 (Table 3). Interestingly, the absolute numbers of cells within specific T-cell subsets that were positively correlated with module 3 were effector/memory, senescent, and activated CD4^+^ and CD8^+^ T-cells (Fig. 3 and Table 3), all subsets known to increase during aging and HIV-1-infection. Naïve CD4^+^ and CD8^+^ T-cells showed a negative correlation (≥0.34) with module 3 (Fig. 3 and Table 3) and no significant correlation with module 3 was seen for B-cell subsets.

**Table 3:** T-cell subsets that correlate with module 3 with a correlation coefficient ≥ 0.34.

Cell Subset	Cell Phenotype	Data Set 1 Correlation with ME3 (p value)	Data Set 2 Correlation with ME3 (p value)
**CD4** ^**+**^ **T-cells:**			
28 negative	CD57^-^CD28^-^CD4^+^	0.40 (0.005)	0.34 (0.02)
	CD45RA^-^CD28^-^CD4^+^	0.40 (0.006)	0.36 (0.01)
Activated	HLADR^+^CD38^+^CD45RO^+^CD4^+^	0.56 (>0.001)	0.59 (>0.001)
	HLADR^+^CD38^+^CD4^+^	0.54 (>0.001)	0.57 (>0.001)
Naïve	CD45RA^+^CD28^+^CD4^+^	-0.40 (0.005)	-0.39 (0.007)
	CD45RA^+^CCR7^+^CD4^+^	-0.41 (0.005)	-0.38 (0.008)
**CD8** ^**+**^ **T-cells**			
Total CD8^+^	CD8^+^CD3^+^	0.44 (0.002)	0.58 (>0.001)
Effector/Memory	CD45RO^+^CD8^+^	0.55 (>0.001)	0.69 (>0.001)
	CD45RA^-^CCR7^-^CD8^+^	0.54 (>0.001)	0.67 (>0.001)
	CD45RA^-^CD28^+^CD8^+^	0.41 (0.004)	0.60 (>0.001)
	CD95^+^CD45RO^+^CD8^+^	0.56 (>0.001)	0.7 (>0.001)
CD28 negative	CD45RA^-^CD28^-^CD8^+^	0.54 (>0.001)	0.59 (>0.001)
	CD57^-^CD28^-^CD8^+^	0.43 (0.003)	0.68 (>0.001)
Early Senescent	CD57^+^CD28^+^CD8^+^	0.42 (0.004)	0.72 (>0.001)
Senescent	CD57^+^CD28^-^CD8^+^	0.48 (>0.001)	0.65 (>0.001)
Activated	HLADR^-^CD38^+^CD45RO^+^CD8^+^	0.42 (0.003)	0.60 (>0.001)
	HLADR^+^CD38^+^CD45RO^+^CD8^+^	0.49 (>0.001)	0.62 (>0.001)
	HLADR^+^CD38^+^CD8^+^	0.52 (>0.001)	0.65 (>0.001)
Naïve	CD45RA^+^CCR7^+^CD8^+^	-0.42 (0.004)	-0.41 (0.004)
	CD95^-^CD45RO^-^CD8^+^	-0.44 (0.002)	-0.45 (0.001)

Included are subsets whose absolute counts have an absolute correlation coefficient with module 3 of ≥ 0.34.

In order to explore whether these effects are due to changes in cellular composition as a result of HIV infection or aging, we applied the RefFreeEWAS algorithm to both of our data sets (S2 and S3 Figs.). This algorithm corrects for changes in cellular composition [45]. The RefFreeEWAS adjusted HIV and aging coefficients were significantly correlated to the unadjusted coefficients for both data sets (S2 Fig.). Also, the reproducibility of both RefFreeEWAS adjusted and unadjusted HIV and aging coefficients significantly correlated between both data sets (S3 Fig.). We also performed a multivariate regression model to determine if the effect of HIV infection on module 3 is only due to changes in cell number of senescent cells, such as CD57^+^CD28^-^CD8^+^ cells. After accounting for the methylation changes resulting from CD57^+^CD28^-^CD8^+^ cells, it appears that some of the effect is due to an increase in this subset, but there is still a significant effect of HIV infection on module 3 (S1 Table).

### Polycomb Group Target genes (PCGT) are highly represented in Module 3

Module eigenvector 3 contains 18,952 CpGs (Table 1). In order to begin the process of identifying the genes that fell within this module, we calculated the module membership value (kME) for each CpG within module 3 using the module eigenvector based connectivity measure[43]. Specifically, for each methylation profile and each module, kME is defined as the correlation of the methylation profile with the module eigenvector. Within the list of 990 CpGs that were found to have a kME>0.85, the most stringent cut-off, there were 14 polycomb group target genes (PCGT) as described by Teschendorff et al. [10] (Table 4). We used this high threshold of 0.85 in order to narrow down the list of intramodular modular hubs to roughly 1000 CpGs (990 exactly), but our results are largely unchanged if other thresholds are chosen. Polycomb group target genes (PCGT) have been shown to be involved in cell renewal, aging and cancer [48–52]. Of note, seven of the PCGT genes in module 3 were represented by multiple different CpG sites that have extremely high kME values. Most striking was *RAB32* which was represented by seven different probes, one of which had the kME value of 0.94, the 3^rd^ highest value of any probe in module 3. Both *PENK* and *GRIA2* were represented in module 3 by three different probes, and a further four genes were represented by two CpG sites (Table 4). The presence of multiple PCGT genes in the module most strongly associated with aging and HIV-1 infection supports a functional association between increased methylation at these sites and aging of the cell.

**Table 4:** Polycomb group target genes (PCGT) represented in module eigenvector 3.

Gene Name	Accession Number	Probe IDs	kME3 of probe*
BNC1	NM_001717	cg04090392	0.88
FBN2	NM_001999	cg05209584	0.90
		cg25084878	0.88
FBX039	NM_153230	cg02093112	0.87
		cg20723355	0.86
GRIA2	NM_001083619	cg22597733	0.87
		cg01942962	0.87
		cg08475096	0.87
HS3ST2	NM_006043	cg03757784	0.88
		cg16399049	0.87
IRX5	NM_005853	cg05266781	0.90
MYOD1	NM_002478	cg20289688	0.86
PENK	NM_001135690	cg04598121	0.88
		cg16219603	0.87
		cg18742346	0.87
RAB32	NM_006834	cg23833452	0.94
		cg01851450	0.89
		cg26252281	0.89
		cg25634742	0.88
		cg01915609	0.88
		cg22030890	0.86
		cg15056556	0.85
SH3GL2	NM_003026	cg17977409	0.87
SIM1	NM_005068	cg04859726	0.86
SLC10A4	NM_152679	cg00967552	0.90
SOX1	NM_005986	cg00663972	0.88
		cg24604013	0.86
SOX8	NM_014587	cg05933904	0.89
TBX5	NM_080717	cg03843000	0.86

*kME is defined as the correlation of the methylation profile with the module eigenvector

## Discussion

These are the first data to show that HIV-1 infection is associated with methylation patterns that are similar to those seen with aging in the general population. Examination of the effects of aging, and the effects of HIV, showed that aging has a stronger effect on changes in methylation. Marginal analysis of the data showed that HIV-1 infection does not have a significant global effect. However, in two separate data sets which significantly correlated with each other, a subset of CpG sites that are hypermethylated with age showed a further increase of methylation levels in individuals infected with HIV-1 (Figs. 1 and 2B). Additionally, a group of sites that show hypomethylation with age are further demethylated in the HIV-1 infected group (Figs. 1 and 2B). Thus, although we have not identified a strong methylation signature associated with HIV-1 infection, our data show that the effects of HIV-1 infection at a subset of methylation sites appear to be additive with the effects of aging.

Multiple studies have shown the effects of HIV-1 infection on the aging of the immune system [15,32–33]. Our own studies on telomere length and decreasing numbers of naïve T-cells showed that individuals with HIV-1 infection appeared immunologically similar to uninfected individuals twenty to thirty years older [32]. It has also been shown that HIV-1 infected individuals develop frailty ten years younger than uninfected individuals [17] and coronary artery calcium measurements show a coronary artery “age” that is accelerated by approximately 15 years with HIV-1 infection [53]. Thus, our data showing HIV-1 acceleration of aging by 13.7 years in data set one and 14.7 years in data set 2, as measured by methylation analysis, fits well with these other studies. As such, it would change if we used other modules or other epigenetic biomarkers of aging [14]. We have focused on module 3 as it showed the strongest positive correlation for both age and HIV-1 status.

Interestingly, we found that absolute numbers of effector/memory, activated, and senescent T-cells in both data sets positively correlated with module 3, the same module that correlated significantly with aging and HIV status. Among the many changes that occur in the immune system with aging, the accumulation of CD28^-^CD8^+^ T-cells, referred to as senescent CD8^+^ T-cells, is strongly correlated with age, reduced vaccine efficacy, increased autoimmunity and the development of aging-related comorbidities such as frailty, bone loss and cardiovascular disease [46–47,54]. Together with decreased T-cell responses to mitogen and reduced B-cell numbers, an inverted CD4^+^/CD8^+^ T-cell ratio and the accumulation of CD28^-^CD8^+^ T-cells was found to be associated with an increased risk of morbidities and mortalities in the OCTO/NONA studies of elderly individuals [55]. In addition to other functional and phenotypic perturbations, these CD28-CD8^+^ T-cells secrete IL-6 and TNF-alpha, thereby directly contributing to the systemic inflammatory environment within the elderly [47]. Inflammaging, the chronic inflammatory environment during aging, is itself associated with damage to organ systems and increased morbidity and mortality. In an intriguing proof-of-principal investigation into the role of senescent T-cells in aging related morbidity, Baker, et al. [56] demonstrated that removal of senescent cells expressing p16^Ink-4a^ from a murine model of Progeria delayed the onset of aging-related diseases in several tissue and organ systems.

HIV-1-infection is thought to contribute to immunosenescence by the activation and expansion of CD8^+^ T-cells directed against both HIV-1 and CMV. The response elicited by HIV-1-infection is also highly inflammatory. Indeed, inflammation during HIV-1-infection is associated with an increased rate of progression to AIDS and mortality [47]. We recently reported that HIV-1-infection was associated with premature accumulation of an immunosenescent phenotype which in turn was associated with faster progression to AIDS [57] leading us to speculate that HIV-1-infection does recapitulate some of the aspects of aging and that these are likely through the inflammatory response and accumulation of senescent cells associated with HIV-1-infection. Some of the changes in methylation in module 3 associated with HIV status can be attributed to a change in numbers of senescent CD57^+^CD28^-^CD8^+^ cells (S1 Table). However, after adjusting for this subset, there was still an effect of HIV status on methylation changes in module 3, suggesting that there are other factors, such as premature aging of naïve T-cells [32], contributing to this effect. Together, this data supports the theory that aging within the immune system plays an important role in aging of the overall organism, and our methylation data suggests that HIV-1 infection may be accelerating this process using similar mechanisms.

We found several Polycomb Group Target Genes represented by CpG’s in module 3. The Polycomb complexes contribute to organismal development by modifying the expression of their targets, the PCGT genes, through epigenetic modulation [58]. Expression of PCGT genes is repressed in stem cells through high levels of methylation as a mechanism of preventing differentiation [58]. Hypermethylation resulting in repressed gene expression is also well established in tumorigenesis (reviewed in [59]). The presence of multiple PCGT genes within the module 3 kME >0.85 group suggests that decreased expression of PCGT genes and the consequent return of cells to less differentiated forms contributes to the increased susceptibility to cancer observed during aging and HIV-1-infection. Most of the PCGT genes in the module 3 kME >0.85 group were represented by more than one CpG site, and, strikingly *RAB32* was represented by seven CpG sites. The fact that seven CpGs associated with *RAB32* show increased methylation strongly suggests that expression of this gene is suppressed in response to aging and HIV-1 infection. RAB32 is a small GTPase related to the oncogene RAS, and is involved in mitochondrial membrane dynamics and apoptosis [60]. Mitochondrial dysfunction is strongly related to aging and cancer [61], and decreased expression of RAB32 may result in disrupted mitochondrial dynamics and changes in apoptotic processes which would also contribute to the development of cancer. Indeed, hypermethylation of RAB32 has been identified in gastric and endometrial cancer [62].

We also evaluated the overlap between our module 3 and the aging module recently described by Horvath, et al. [12]. The degree of overlap with the aging module, found in human brain tissues and other solid tissues [12] is highly significant (Fisher-exact p-value = 5.6 x 10^-6^, odds ratio = 1.91). Interestingly, one of the overlapping genes represented by both modules (the aging module and our module 3) is *RAB32*, which we discussed above. These results strongly suggest that our module 3 is not specific to blood tissue. Future research should evaluate whether module 3 also reveals HIV related age acceleration effects in other solid tissues. Taken together, these data suggest that HIV-1-infection does accelerate some aspects of aging and that general aging and HIV-1 related aging work through at least some common mechanisms. These results are an important first step for finding potential therapeutic approaches to mitigate the effects of both HIV and aging.

## Supporting Information

S1 Fig Reproducibility of HIV and CpG-methylation Associations between Data Sets 1 and 2. CpG methylation was tested for the association with HIV-status by an unpaired t-test. One percent of all applicable CpG probes were randomly sampled and the corresponding t-values for data set 1 and 2 were used for representative plotting. The t-values between data sets were found to have a Pearson correlation of 0.39 (p < 10^-200^). The black line represents the identity of t-values between datasets.
(EPS)

S2 Fig Effects of RefFreeEWAS-adjustment on HIV and Age Coefficients within Data Sets. Reference Free EWAS models were generated using HIV status and age as covariates. The unadjusted and adjusted coefficients were correlated and 1% of all CpGs were randomly sampled. The corresponding coefficients were used for representative plotting within each dataset. The black lines represent the identity of coefficients before and after RefFreeEWAS adjustment.
(EPS)

S3 Fig Reproducibility of HIV and Age Coefficients between Data Sets before and after RefFreeEWAS-adjustment. Reference Free EWAS models were generated using HIV status and age as covariates. Pearson’s correlation was computed between data sets 1 and 2 for before and after adjustment. Representative plots were generated by randomly sampling one percent of applicable CpGs and plotting their corresponding covariate coefficients for comparison between data sets. The black lines represent the identity of the coefficients between datasets.
(EPS)

S1 Table Multivariate regression model determining contribution of CD57^+^CD28^-^CD8^+^ T cells to differences in methylation with HIV infection. (DOCX)
