# Supplementary material for: Acceleration of Age-Associated Methylation Patterns in HIV-1-Infected Adults
Source: PLoS One. 2015 Mar 25;10(3):e0119201. doi: 10.1371/journal.pone.0119201 (PMC4373843; doi:10.1371/journal.pone.0119201)
Supplement: Supplementary file 1 [file pone.0119201.s004.docx]

S1 Table. Multivariate regression model determining contribution of CD57+CD28-CD8+ T cells to differences in methylation with HIV infection.
